# Investigation of the relationship between uterine artery Doppler diastolic notching and serum apelin-13 and apelin-36 concentrations between the 11^th^ and 14^th^ weeks of pregnancy: a case-control study

**DOI:** 10.11604/pamj.2025.52.82.44289

**Published:** 2025-10-24

**Authors:** Fethiye Demirtaş Acar, İbrahim Kale, Murat Muhcu

**Affiliations:** 1 Umraniye Training and Research Hospital, Department of Obstetrics and Gynecology, Istanbul, Turkey

**Keywords:** Apelin-13, apelin-36, diastolic notching, pregnancy, uterine artery

## Abstract

**Introduction:**

the apelin family plays a role in the proliferation and migration of trophoblasts. Based on this, we aimed to investigate the relationship between diastolic notching in uterine artery Doppler and serum apelin-13 and apelin-36 concentrations between 11 and 14 weeks of gestation.

**Methods:**

forty-four (44) pregnant women with diastolic notching in uterine artery Doppler and 44 without diastolic notching were compared in terms of serum apelin-13 and apelin-36 concentrations.

**Results:**

the two groups were similar in terms of demographic characteristics (p > 0.05). The median apelin-13 concentration was found to be 45.4 pg/mL in the uterine artery notch-positive group, while it was 41.02 pg/mL in the uterine artery notch-negative group (p = 0.770). The median apelin-36 concentration was 546.06 pg/mL in the uterine artery notch-positive group, while it was 536.6 pg/mL in the uterine artery notch-negative group (p = 0.570). Participants were regrouped as high or normal pulsatility index (PI) according to uterine artery PI, and both groups were compared in terms of apelin-13 and apelin-36 concentrations. Median serum apelin-13 and apelin-36 concentrations were also similar in the high and normal uterine artery PI groups (p = 0.445, p = 0.402, respectively).

**Conclusion:**

although the number of participants was too small to draw a definitive conclusion, no significant relationship between diastolic notching or increased resistance in the uterine artery and serum apelin-13 and apelin-36 concentrations could be detected.

## Introduction

During the early weeks of pregnancy, the vascular walls of the spiral arteries undergo a series of complex morphological changes due to invasion by trophoblasts. At the end of this physiological process, the spiral arteries turn into larger vessels with low resistance and high compliance, which do not respond to the mother's vasomotor activity. This vascular change in the spiral arteries is essential to provide adequate blood flow to the growing fetus in the uterus [1,2]. Uterine artery Doppler has been used to evaluate placental flow dynamics during pregnancy for many years. Doppler ultrasound of the uterine artery in early pregnancy typically demonstrates slow end-diastolic velocities and an early diastolic notch [3]. Notching in the uterine artery Doppler waveform is observed in 46-64% of pregnancies in the first trimester [4]. Also, studies demonstrated that in uncomplicated pregnancies, the diastolic notching in the uterine arteries disappears by the 26^th^ week of gestation [5,6]. Apelin is an endogenous ligand of the G protein-coupled receptor (APJ, angiotensin II receptor-like 1) on the cell surface. Apelin originates from a precursor peptide consisting of 77 amino acids that is converted to active fragments (such as apelin-10, apelin-11, apelin-12, apelin-13, apelin-15, apelin-17, apelin-19, and apelin-36) [7].

All apelin fragments exert their effects through the APJ receptor. However, among these fragments, apelin-13 is the most biologically active fragment, while apelin-36 has the highest binding affinity for the APJ receptor [8]. The apelin and its receptor APJ are expressed in the endothelial cells of many vessels, adipose tissue, and placenta [9,10]. As a physiological effect of the apelin/APJ system, it has been shown to reduce blood pressure in rats through a nitric oxide-dependent mechanism [11]. A study published in 2020 suggested that apelin, which promotes trophoblast cell proliferation through APJ and ERK1/2, Stat3, and AMPKα signaling, is a crucial adipokine in regulating early placental development [12]. A different study published in 2023 suggested that apelin-36 could alleviate LPS-induced cell inflammation and apoptosis and improve the invasion and migration of trophoblasts by inhibiting the GRP78 /ASK1/JNK signaling [13]. We thought that apelin, which provides vasodilatation through a nitric oxide-dependent mechanism and plays a role in the survival, proliferation, and migration of trophoblasts, might be associated with the formation of a diastolic notch in uterine artery Doppler. Based on this, we aimed to investigate serum apelin-13 and apelin-36 concentrations in pregnant women with and without a diastolic notch in uterine artery Doppler between the 11^th^ and 14^th^ weeks of pregnancy. We hypothesized that serum apelin-13 and apelin-36 concentrations would be lower in the group with a notch in the uterine artery Doppler than in the group without a notch.

## Methods

**Study design and setting:** this prospective, non-interventional case-control study was conducted with 88 pregnant women aged between 18 and 39, who applied to Umraniye Training and Research Hospital, Department of Obstetrics and Gynecology, Istanbul, Turkey, for a first-trimester screening test between March 2023 and October 2023.

**Participants:** forty-four (44) singleton pregnant women with unilateral or bilateral diastolic notching detected in uterine artery Doppler between the 11^th^ and 14^th^ weeks of pregnancy formed the study group (uterine artery notch-positive group). Forty-four (44) singleton pregnant women who did not have diastolic notching in uterine artery Doppler between the 11^th^ and 14^th^ weeks of pregnancy constituted the control group (uterine artery notch-negative group). The uterine artery notch-positive and notch-negative groups were formed by matching age, body mass index (BMI), parity, and gestational age at which the blood sample was taken. Smokers, pregnant women who conceived with assisted reproductive techniques, pregnant women with multiple pregnancies, and pregnant women who started with multiples and continued with singletons were not included in the study. Pregnant women with any pregestational disease, thrombophilia, or congenital uterine anomaly, and pregnant women with a history of recurrent pregnancy loss were not included in the study. Pregnant women using aspirin or heparin were not included in the study.

**Data sources and measurements:** participants' demographic characteristics and obstetric histories were noted. Fetal crown-rump length (CRL), nuchal translucency (NT), and uterine artery Doppler measurements were made by the same perinatologist using the wide-band convex probe (maximum bandwidth: 2-8MHz, maximum depth: 26 cm) of the GE Voluson E6 Ultrasound System (GE HealthCare, Chicago, IL, USA). Doppler pulsatility index (PI), resistance index (RI), and systole/diastole ratio (S/D) of both uterine arteries were measured separately, and the average value was calculated. The presence of an early diastolic notch in the unilateral or bilateral uterine artery was noted.

**Laboratory analysis:** after ultrasonographic examination, venous blood samples were taken from the participants to measure serum apelin-13 and apelin-36 concentrations. The samples were then centrifuged at 3000 rpm for 20 min. After centrifugation, the supernatant remaining on the upper part of the tube was transferred into an Eppendorf tube and stored at -80°C. Apelin-13 concentrations in blood samples were measured using the Fine Test Human Apelin-13 Kit (Wuhan Fine Biotech Co., Ltd., Wuhan, Hubei, China; Catalogue No: EH2649) by the enzyme-linked immunosorbent assay (ELISA) method. For the Human Apelin-13 Kit used in the study, a measurement value between 125 - 8000 pg/mL and a sensitivity of 75 pg/mL was determined. For the apelin-13 Kit, the intra-assay coefficient of variation was < 8% and the inter-assay coefficient of variation was < 10%. Apelin-36 concentrations in blood samples from participants were measured using the Fine Test Human Apelin-36 Kit (Wuhan Fine Biotech Co., Ltd., Wuhan, Hubei, China, Catalogue No: EH1971) by the ELISA method. For the Human Apelin-36 Kit used in the study, a measurement value between 46.875- 3000 pg/mL and a sensitivity of 28.125 pg/mL was determined. For the Apelin-36 Kit, the intra-assay coefficient of variation was < 8% and the inter-assay coefficient of variation was < 10%.

**Study size:** a power analysis was performed using the G*Power software (version 3.9.4) to determine the sample size. The statistical power of a study is expressed as 1-β (where β represents the probability of a Type II error). Scientific studies are generally expected to have a power of 80% at a significance level of α=0.05. Based on the differences in serum apelin-13 concentrations between the preeclampsia and control groups reported by Gürlek *et al*.in their study entitled “Evaluation of serum apelin-13 and apelin-36 concentrations in preeclamptic pregnancies,” the required sample size was calculated [14]. This calculation required 40 participants per group to achieve 80% power at α=0.05. Considering potential dropouts during the study period, it was planned to include 44 participants in each group. Since no participants were lost during the study, the final analysis was conducted on 88 participants, 44 in the uterine artery notch-positive group and 44 in the uterine artery notch-negative group.

**Statistical methods:** statistical analysis was performed with Statistical Package for the Social Sciences (SPSS) version 25.0 (IBM Corp., Chicago, IL, USA). The Kolmogorov-Smirnov test was used to check whether the data were normally distributed. Descriptive statistical methods (mean, standard deviation, median, 25% quartile, 75% quartile, frequency, and ratio) were used when evaluating the study data. An independent t-test was used to compare two groups showing parametric distribution, and the Mann-Whitney U test was used to compare two groups showing non-parametric distribution. One-way ANOVA was used to compare three groups showing parametric distribution, and the Kruskal-Wallis test was used to compare more than two groups showing non-parametric distribution. Correlation analysis was used to analyze the relationship between quantitative data, and the Chi-square test was used to analyze categorical data. Statistical significance was accepted as p < 0.05 for all values.

**Ethical considerations of consent:** Istanbul Umraniye Training and Research Hospital Local Ethics Committee approved this study (Ethics Committee Approval Number: B.10.1.TKH.4.34.H.GP.0.01/31, Date: 30/01/2023). The Declaration of Helsinki maintained the study protocol. Informed and written consent was obtained from all participants.

**Data availability:** the data sets created and analyzed during the current study are not publicly available due to the rules of the Istanbul Umraniye Training and Research Hospital Local Ethics Committee, but can be obtained from the corresponding author upon reasonable request.

## Results

**Characteristics of sample 1:** forty-four (44) singleton pregnant women with unilateral or bilateral diastolic notching in the uterine artery and 44 singleton pregnant women without diastolic notching in the uterine artery were compared in terms of demographic features, ultrasound measurements, and laboratory test results. Both groups were similar in terms of age, BMI, gravida, parity, presence of polycystic ovary syndrome (PCOS), history of preeclampsia, and history of fetal growth restriction (FGR) (p > 0.05, for all) (Table 1). Both groups were similar in terms of fetal CRL measurement, NT measurement, NT MoM value, gestational week at blood sampling, PAPP-A level, PAPP-A MoM value, human chorionic gonadotropin (hCG) level, and hCG MoM value (p > 0.05, for all). Mean uterine artery PI, RI, and S/D were significantly higher in the uterine artery notch-positive group than in the notch-negative group (p < 0.001, p = 0.002, p = 0.003, respectively). The median apelin-13 concentration was found to be 45.4 pg/mL in the uterine artery notch-positive group, while it was 41.02 pg/mL in the uterine artery notch-negative group (p = 0.770). The median apelin-36 concentration was 546.06 pg/mL in the uterine artery notch-positive group, while it was 536.6 pg/mL in the uterine artery notch-negative group (p = 0.570) (Table 2).

**Table 1:** comparison of the uterine artery notch-negative and notch-positive groups in terms of demographic features

		Uterine artery notch-negative group (n = 44)	Uterine artery notch-positive group (n = 44)	
		Mean ± SD Median (%25 - % 75)	Mean ±SD Median (%25 - % 75)	P-value
**Age (Years)**		29.5 ± 4.92 30 (26 - 32.5)	27.5 ± 4.4 27 (24 - 30)	0.053*
**BMI (kg/m^2^)**		24.9 ± 2.7 25.3 (22.6 - 26.8)	24.2 ± 3.3 24.7 (21 - 26.8)	0.326**
**Gravida**		2.2 ± 1.3 2 (1 - 3)	2 ± 1.2 2 (1 - 3)	0.386**
		**n (%)**	**n (%)**	
**Parity**	**Nulliparous**	19 (43.2)	20 (45.5)	1.000***
	**Multiparous**	25 (56.8)	24 (54.5)	
**Presence of PCOS**		2 (4.5)	2 (4.5)	1.000***
**History of Preeclampsia**		0 (0)	2 (4.5)	0.494***
**History of FGR**		1 (2.3)	2 (4.5)	1.000***

* Independent t test; **Man-Whitney U test; ***Chi-Square test; BMI: body mass index; PCOS: polycystic ovary syndrome; FGR: fetal growth restriction

**Table 2:** comparison of uterine artery notch-negative and notch-positive groups in terms of ultrasonographic measurements and laboratory results

	Uterine artery notch-negative group (n = 44)	Uterine artery notch-positive group (n = 44)	
	Mean ± SD Median (%25 - %75)	Mean ± SD Median (%25 - %75)	P-value
**CRL (mm)**	62.2 ± 10.39 61.5 (54.5- 69.5)	62.64 ± 9.24 62.5 (55.5 – 67)	0.835*
**NT (mm)**	1.51 ± 0.41 1.51 (1.2-1.86)	1.56 ± 0.4 1.55 (1.28 - 1.77)	0.557*
**NT MoM**	0.95 ± 0.23 0.95 (0.78 - 1.04)	0.97 ± 0.19 0.93 (0.8-1.09)	0.626*
**Mean uterine artery PI**	1.6 ± 0.42 1.54 (1.28 - 1.9)	2.03 ± 0.51 1.92 (1.63 - 2.32)	< 0.001*
**Mean uterine artery RI**	0.74 ± 0.09 0.74 (0.69 - 0.81)	0.8 ± 0.08 0.8 (0.75-0.86)	0.002*
**Mean Uterine Artery S/D**	4.54 ± 1.58 4.21 (3.33 -5.61)	6.55 ± 3.72 5.23 (4.26-7.56)	0.003**
**Gestational week at blood sampling**	12 ± 0.7 12 (11-13)	12 ± 0.7 12 (12 - 13)	0.676**
**PAPP-A level (mlU/mL)**	3.35 ± 1.98 2.62 (1.9 - 4.62)	4.52 ± 5.05 3.34 (2.24 ± 4.98)	0.282**
**PAPP-A MoM**	1.08 ± 0.57 0.98 (0.54 - 1.51)	1.39 ± 0.06 1 (0.79-1.34)	0.689**
**hCG level (ng/mL)**	32.88 ± 18.04 29.2 (18.53- 42.35)	43.72 ± 37.65 30.6 (19.1-53.74)	0.582**
**hCG MoM**	0.89 ±0.48 0.75 (0.56-1.23)	1.1 ± 0.86 0.75 (0.5-1.51)	0.726**
**Apelin-13 concentration (pg/mL)**	78.38 ± 79.63 41.02 (35.97- 83.47)	66.67 ± 51.21 45.4 (32.47-87.94)	0.770**
**Apelin-36 concentration (pg/mL)**	975.4 ± 941.92 536.6 (414.4 -1267)	885.36 ± 847.1 546.06 (373.80- 936.81)	0.570**

* Independent t Test; ** Man-Whitney U Test; CRL: crown rump length; PI: pulsatility index; RI: resistance index; S/D: systolic/diastolic Ratio; NT: nuchal translucency; PAPP-A: pregnancy-associated plasma protein-A; hCG: human chorionic gonadotropin

**Subgroup analysis:** the uterine artery notch-positive group was divided into two subgroups: those with the unilateral notch and those with the bilateral notch. These subgroups were compared with the uterine artery notch-negative group. Three groups were similar in terms of fetal CRL measurement, NT measurement, NT MoM value, gestational week at blood sampling, PAPP-A level, PAPP-A MoM value, hCG level, and hCG MoM value (p > 0.05, for all). Uterine artery PI and RI were higher in the bilateral uterine artery notch-positive group than in the uterine artery notch-negative group (p < 0.001, p = 0.001, respectively). Three groups were similar in terms of uterine artery S/D (p = 0.231). While the median apelin-13 concentration was found to be 41.02 pg/mL in the uterine artery notch-negative group, it was 42.60 pg/mL in the unilateral uterine artery notch-positive group and 45.65 pg/mL in the bilateral uterine artery notch-positive group (p = 0.472). The median apelin-36 concentration was found to be 536.6 pg/mL in the uterine artery notch-negative group, while it was 476.76 pg/mL in the unilateral uterine artery notch-positive group and 627.19 pg/mL in the bilateral uterine artery notch-positive group (p = 0.183) (Table 3).

**Table 3:** comparison of uterine artery notch-negative, unilateral uterine artery notch-positive, and bilateral uterine artery notch-positive groups in terms of ultrasonographic measurements and laboratory results

	Uterine artery notch-negative group1 (n = 44)	Unilateral uterine artery notch-positive group 2 (n = 16)	bilateral uterine artery notch-positive group 3 (n = 28)	p-value	Post-Hoc
	Mean ± SD Median (%25-%75)	Mean ± SD Median (%25-%75)	Mean ± SD Median (%25-%75)		
**CRL (mm)**	62.2 ± 10.39 61.5 (54.5 - 69.5)	63.26 ± 7.03 65 (60 - 67)	62.16 ± 10.74 62 (55- 67)	0.800*	
**NT (mm)**	1.51 ± 0.41 1.51 (1.2-1.86)	1.63 ± 0.42 1.7 (1.25-1.89)	1.51 ± 0.38 1.43 (1.3-1.7)	0.540*	
**NT MoM**	0.95 ±0.23 0.95 (0.78-1.04)	0.99 ± 0.2 1.01 (0.8-1.15)	0.95 ± 0.19 0.89 (0.82-1.05)	0.682*	
**Mean Uterine Artery PI**	1.6 ± 0.42 1.54 (1.28 - 1.9)	1.87 ± 0.4 1.84 (1.52- 2.06)	2.15 ± 0.56 1.96 (1.69 -2.49)	< 0.001*	3>1 (p < 0.001)
**Mean Uterine Artery RI**	0.74 ± 0.09 0.74 (0.69-0.81)	0.78 ± 0.07 0.77 (0.73-0.83)	0.82 ±0.09 0.81 (0.76-0.89)	0.002*	3>1 (p< 0.001)
**Mean Uterine Artery S/D**	4.54 ± 1.58 4.21 (3.33- 5.61)	5.19 ± 2.27 4.87 (3.77- 5.72)	7.58 ± 4.28 6.32 (4.55-10.13)	0.231**	
**Gestational week at blood sampling**	12 ± 0.7 12 (11-13)	12.4 ± 0.6 12.2 (11.9-12.9)	12.1 ± 0.7 12.0 (11.7 -12.7)	0.549*	
**PAPP-A level (mlU/mL)**	3.35 ± 1.98 2.62 (1.9-4.62)	5.63 ± 7.13 3.81 (2.84-4.71)	3.68 ± 2.44 2.57 (2.01- 5.04)	0.123**	
**PAPP-A MoM**	1.08 ± 0.57 0.98 (0.54-1.51)	1.9 ± 3.06 1.14 (0.79-1.63)	1.01 ± 0.41 0.96 (0.79-1.25)	0.338**	
**hCG level (ng/mL)**	32.88 ± 18.04 29.2 (18.53-42.35)	36.74 ± 34.26 24.35 (15.74- 49.36)	49.02 ± 39.88 31.19 (21.44- 68.86)	0.621**	
**hCG MoM**	0.89 ± 0.48 0.75 (0.56-1.23)	0.96 ± 0.85 0.71 (0.37-1.18)	1.2 ±0.86 0.77 (0.59-1.67)	0.590**	
**Apelin-13 concentration (pg/mL)**	78.38 ± 79.63 41.02 (35.97- 83.47)	59.48 ± 42.78 42.60 (28.66- 72.30)	72.14 ± 57.04 45.65 (33.98-93.90)	0.472**	
**Apelin-36 concentration (pg/mL)**	975.4 ± 941.92 536.6 (414.4-1267)	678.58± 576.94 476.76 (336.34- 669.61)	1042.52 ± 988.15 627.19 (401.7-1563.2)	0.183**	

* One Way ANOVA Test; **Kruskal wallis test; CRL: crown rump length; PI: pulsatility index; RI: resistance index; S/D: systolic/diastolic ratio; NT: nuchal translucency; PAPP-A: pregnancy-associated plasma protein-A; HCG: human chorionic gonadotropin

**Characteristics of sample 2:** participants were grouped as high PI (PI ≥ 2.3) (n = 14) or normal PI (PI < 2.3) (n = 74) according to uterine artery PI. Both groups were similar in terms of fetal CRL measurement, NT measurement, NT MoM value, gestational week at blood sampling, PAPP-A level, PAPP-A MoM value, hCG level, and hCG MoM value (p > 0.05, for all). Mean uterine artery PI, RI, and S/D were significantly higher in the high uterine artery PI group than in the normal uterine artery PI group (p < 0.001, for all). The median apelin-13 concentration was 41.85 pg/mL in the high uterine artery PI group, while 42.18 pg/mL in the normal uterine artery PI group (p = 0.445). The median apelin-36 concentration was 479.99 pg/mL in the high uterine artery PI group, while it was 544.79 pg/mL in the normal uterine artery PI group (p = 0.402) (Table 4).

**Table 4:** comparison of high and normal uterine artery PI groups in terms of ultrasonographic measurements and laboratory results

	Normal uterine artery PI group (PI < 2.3) (n = 74)	High uterine artery PI group (PI ≥ 2.3) (n = 14)	
	Mean ± SD Median (%25-%75)	Mean ± SD Median (%25-%75)	P-value
CRL (mm)	63.19 ± 9.82 63 (56 -70)	58.36± 8.76 56 (52- 67)	0.090*
NT (mm)	1.55 ± 0.39 1.56 (1.21-1.8)	1.48 ± 0.47 1.4 (1.14 -1.56)	0.540*
NT MoM	0.96 ± 0.21 0.96 (0.8- 1.05)	0.96 ± 0.24 0.89 (0.8-1.11)	0.927*
Mean uterine artery PI	1.65± 0.34 1.65 (1.43-1.92)	2.71 ±0.29 2.65 (2.51- 2.8)	< 0.001*
Mean uterine artery RI	0.74 ± 0.08 0.76 (0.7-0.8)	0.89 ± 0.03 0.9 (0.86- 0.91)	< 0.001*
Mean uterine artery S/D	4.56 ± 1.39 4.4 (3.56- 5.59)	10.76 ± 3.9 10.21 (8.47-12.17)	< 0.001**
Gestational week at blood sampling	12 ± 0.7 12 (12-13)	11.8 ±0.7 12 (11-12)	0.328**
PAPP-A level (mlU/mL)	3.97 ± 4.11 3.01 (1.99-4.84)	3.75 ± 2.17 3.18 (2.26- 4.92)	0.758**
&PAPP-A MoM	1.24 ± 1.63 0.98 (0.59 -1.41)	1.2 ± 0.46 1.1 (0.93-1.63)	0.283**
hCG level (ng/mL)	36.3± 29.57 28.52 (18.21± 44.87)	48.84 ± 30.17 36.16 (27.2-68.86)	0.071**
hCG MoM	0.96 ± 0.7 0.73 (0.51-1.18)	1.19 ± 0.66 1.06 (0.7-1.67)	0.106**
Apelin-13 concentration (pg/mL)	75.21 ± 69.88 42.18 (34.59-83.06)	58.35 ± 46.87 41.85 (28.38 -92.82)	0.445**
Apelin-36 concentration (pg/mL)	937.28 ± 872.2 544.79 (406.12-1063.44)	893.90± 1023.90 479.99 (358.61-1028.33)	0.402**

* Independent t Test; ** Man-Whitney U Test; CRL: crown rump length, PI: pulsatility index; RI: resistance index, S/D: systolic/diastolic ratio; NT: nuchal translucency; PAPP-A: pregnancy-associated plasma protein-A; hCG: human chorionic gonadotropin

## Discussion

This study investigated the relationship between uterine artery diastolic notching and serum apelin-13 and apelin-36 concentrations during 11-14 weeks of gestation. Serum apelin-13 and apelin-36 concentrations were similar in the uterine artery notch-positive and notch-negative groups. The pathophysiology of the uterine artery diastolic notch is not yet clearly understood. It has been tried to be explained with some theories. A study published in 1988 suggested that the uterine artery's diastolic notch resulted from wave reflection due to high placental bed resistance [15]. In 1995, Talbert *et al*. proposed that the uterine artery diastolic notch was caused by increased arterial wall compliance [16]. In a separate study, Brodszki *et al*. reported that uterine artery notching was not associated with changes in the mechanical properties of large vessels, as assessed by ultrasound, but rather resulted from endothelial dysfunction accompanied by alterations in serum nitrite and nitrate levels [17]. Finally, Kim *et al*. stated that the uterine artery notch is associated with increased placental endothelial nitric oxide synthase and heat shock protein [18]. Apelin is a pleiotropic peptide whose primary function is cardiovascular regulation and body fluid homeostasis [19,20]. Due to this feature, the role of apelin in the pathophysiology of preeclampsia has been investigated in previously published studies, and conflicting results have been reported.

Different studies show that serum apelin levels of pregnant women with preeclampsia are lower, higher, or similar to those of normotensive pregnant women [21-23]. A study published in 2023 showed that serum apelin concentration increased in pregnant women with preeclampsia, but APJ expression decreased in preeclamptic placentas. Molecular examination of the placentas revealed that the Apelin/APJ system protects trophoblasts from hypoxia-induced oxidative stress by activating the PI3K/Akt signaling pathway in preeclampsia [24]. In 2020, a paper was published investigating the expression of apelin and its receptor in the placenta, its role in the signaling pathway, and trophoblast regulation. The apelin expression seemed to be higher in cytotrophoblast cells than in syncytiotrophoblasts. Also, apelin has been shown to increase cell proliferation by modulating cell cycle progression by affecting the activation of cyclins. In addition, it was observed that apelin stimulates placenta cell proliferation via APJ activation and kinases ERK1/2, Stat3, and AMPKα pathways. As a result of this study, the authors suggested that apelin is an important adipokine for proper placental development in early pregnancy [12]. In addition to modulating the proliferation of trophoblast cells, apelin has also been shown to have an anti-apoptotic effect on the placenta. The anti-apoptotic effect of apelin was mediated by the APJ receptor and mitogen-activated protein kinase (ERK1/2/MAP3/1) and protein kinase B (AKT). With these results, the authors again suggested the important involvement of apelin in early placental development [25]. These results were also supported by another study published in 2023. Accordingly, it was shown that apelin-36 can alleviate lipopolysaccharide-induced cell inflammation and apoptosis and improve the invasion and migration of trophoblasts by inhibiting the GRP78/ASK1/JNK signaling [13].

To our knowledge, this is the first study in the literature to examine the relationship between serum apelin-13 and apelin-36 concentrations and notching in the uterine artery. At the beginning of this study, we thought that serum apelin-13 and apelin-36 might contribute to uterine artery diastolic notching formation. We hypothesized that apelin-13 and apelin-36 concentrations would be lower in the group with a diastolic notch on the uterine artery than in the group without a notch. However, we did not detect a significant relationship between diastolic notching or resistance increase in the uterine artery and serum apelin-13 and apelin-36 concentrations.

**Study limitations:** the small number of participants, the fact that serum apelin-13 and apelin-36 concentrations were measured only once, and the perinatal outcomes of the participants were not followed the important limitations of this single-center study.

## Conclusion

No relationship between diastolic notching in the uterine artery and serum apelin-13 and apelin-36 concentrations between 11 and 14 weeks of gestation could be detected. Additionally, no relationship was detected between high uterine artery Doppler resistance and serum apelin-13 and apelin-36 concentrations in the same gestational weeks. However, it should not be forgotten that the number of participants in this study was relatively small to draw a definitive conclusion.

### What is known about this topic

- The exact cause of the diastolic notching observed on uterine artery Doppler is still unknown;
- The Apelin family plays a role in the survival, proliferation, and migration of trophoblasts.

### What this study adds

- No relationship was detected between diastolic notching in the uterine artery and serum apelin-13 and apelin-36 concentrations between 11 and 14 weeks of gestation;
- No relationship was detected between high uterine artery Doppler resistance and serum apelin-13 and apelin-36 concentrations between 11 and 14 weeks of gestation.
